# Supplementary material for: Single-cell RNA analysis reveals unexpected hemocyte plasticity and immune cell specialization in a Drosophila overgrowth model
Source: bioRxiv. 2026 Jan 11:2026.01.09.698745. Preprint. [Version 1] doi: 10.64898/2026.01.09.698745 (PMC12803228; doi:10.64898/2026.01.09.698745)

Fig S1: Quality control and Specicity of Hemocyte preparation for Sc-seq data analysis

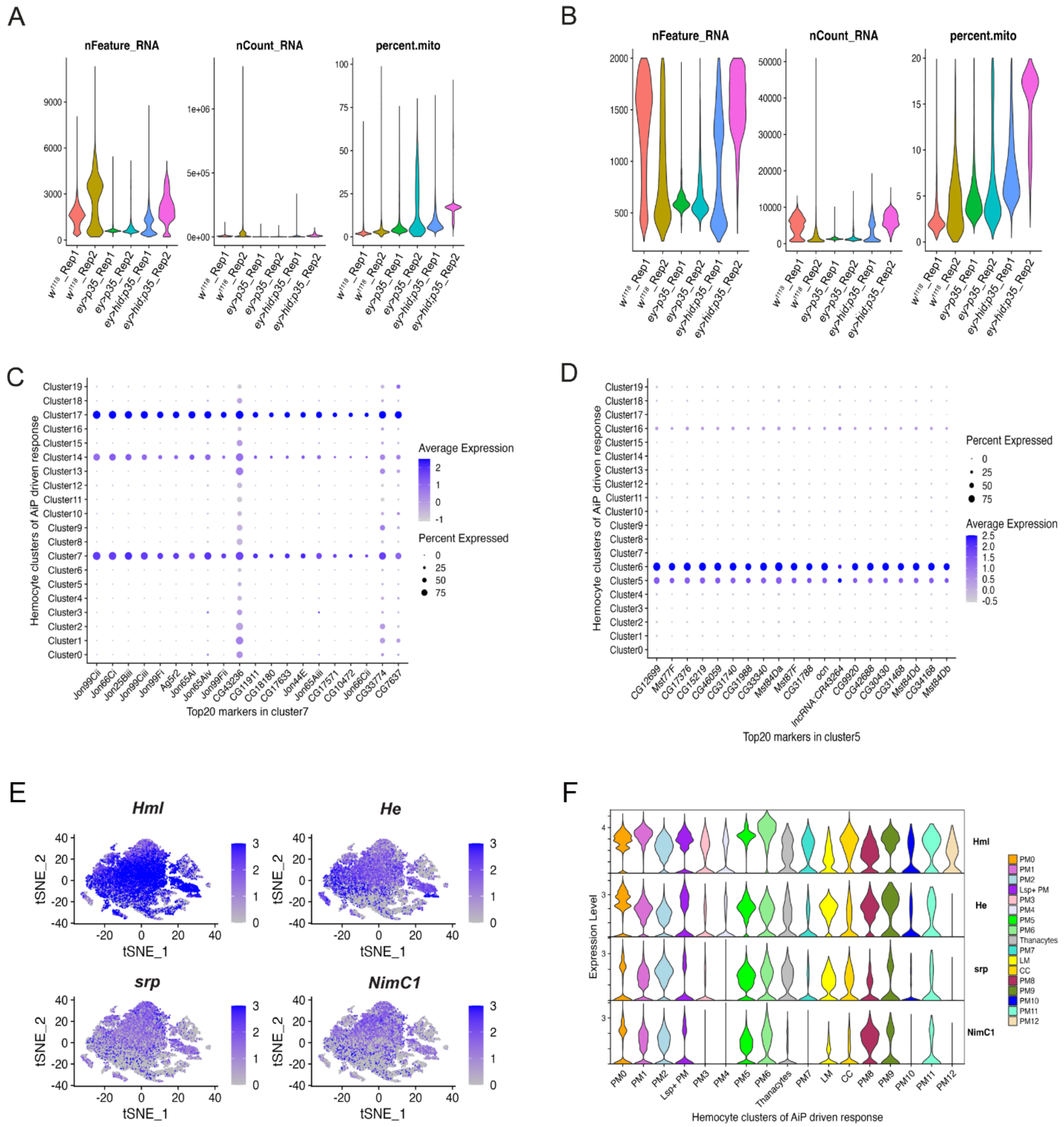

Fig S2: Identification of known hemocyte types

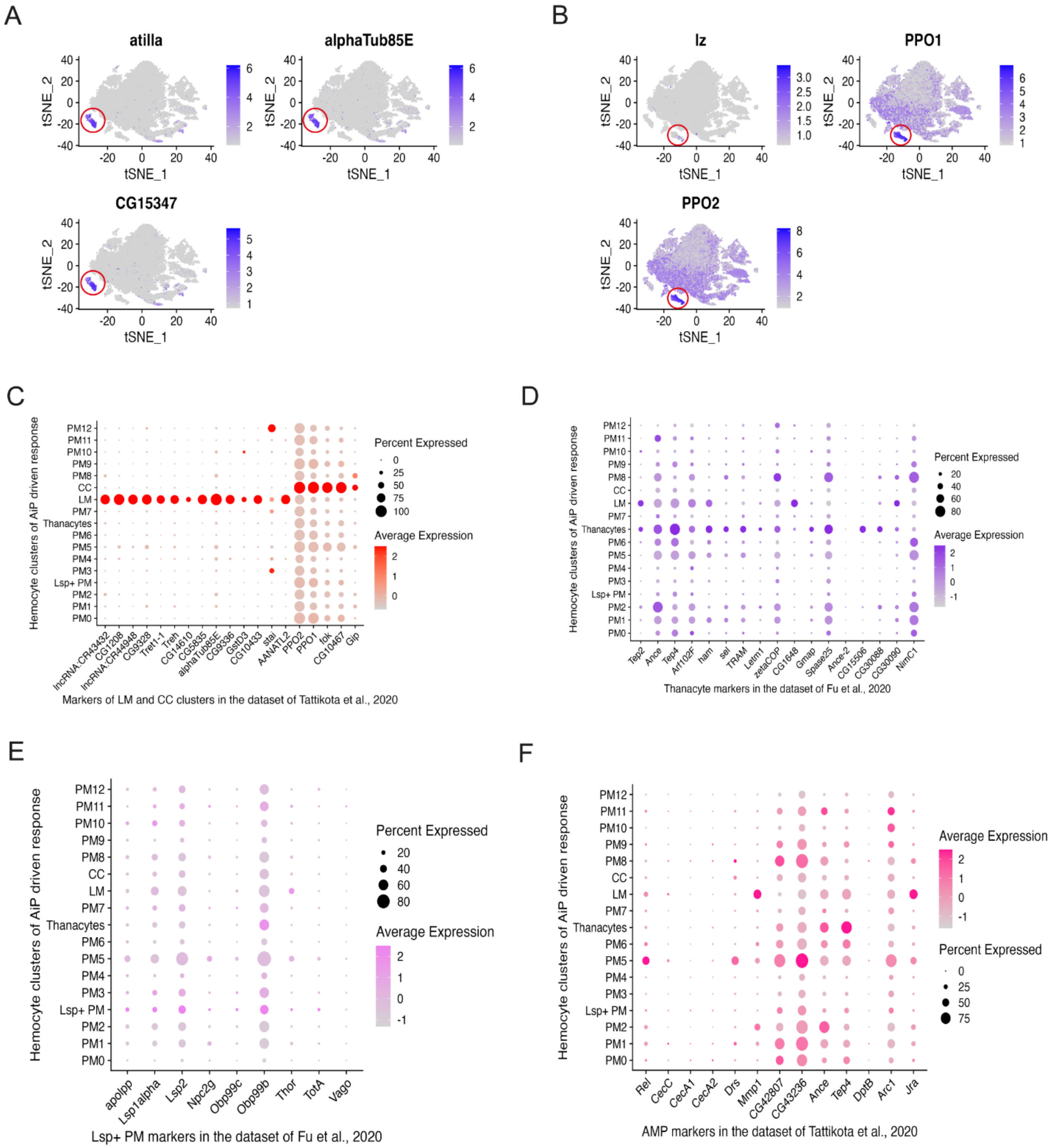

Fig S3: GO Analysis of all new plasmatocyte clusters

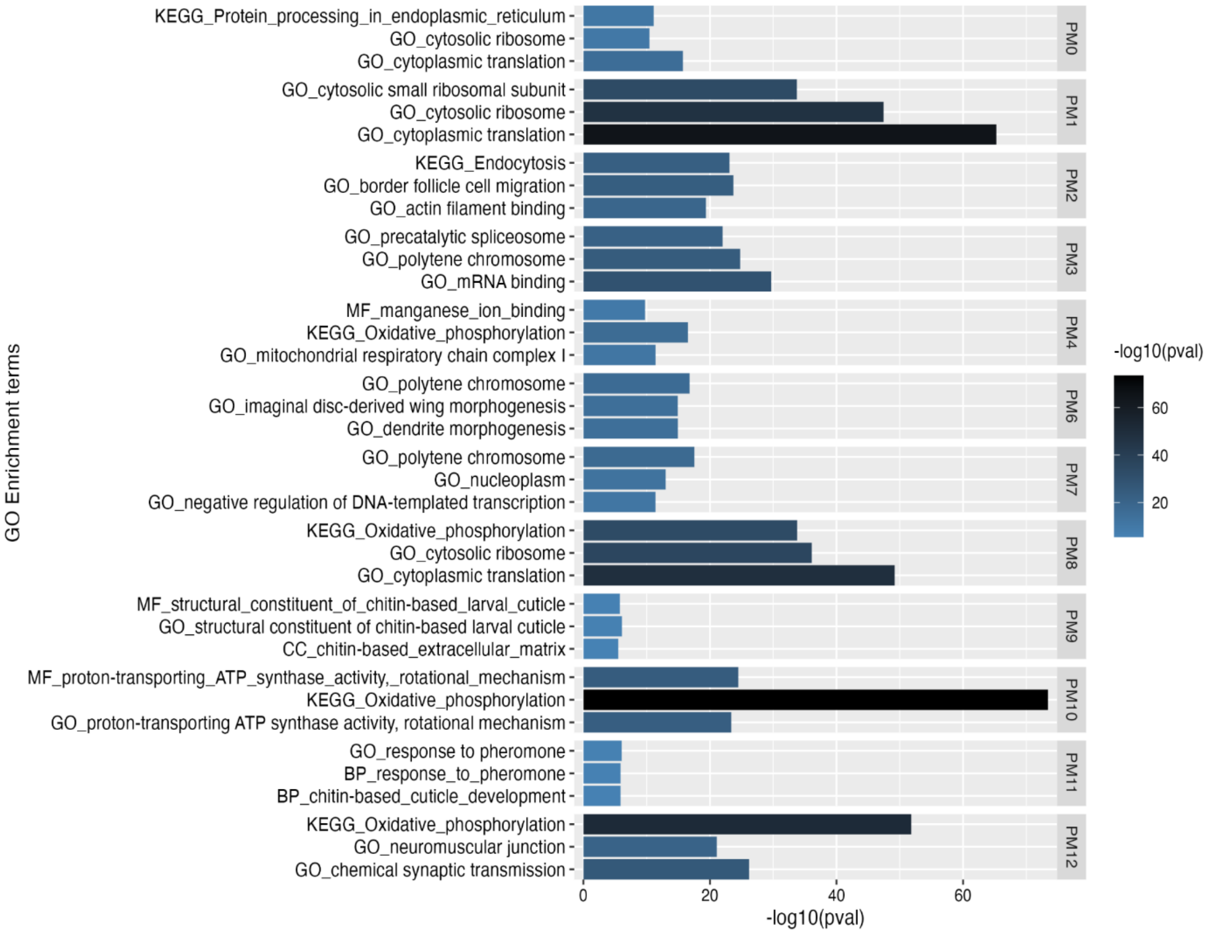

Supplement: Supplement 1 — Supplementary Figure S1. Quality control and validation of scRNA-seq datasets from hemocytes (A) Distribution of detected transcripts, mitochondrial gene percent and number of transcript variants per each sample from the analysis. (B) Violin plots showing quality filters set from Fig S1A. Each sample containing ~2000 genes per cell along with median transcript content and ≤20% mitochondrial gene content were retained for downstream analysis. (C) Dot plot displaying expression of key marker genes of clusters 7, 14 and 17 indicating transcriptional overlap between initially identified clusters. Clusters 7, 14 and 17 sharing similar marker profiles were merged into a single cluster, yielding PM5 in the final integrated dataset. Color gradient indicates the expression level while the size of the dot represents percentage of cells showing the expression. (D Dot plot displaying expression of key marker genes of clusters 5 and 6 showing transcriptional overlap between initially identified clusters. Clusters 5 and 6 sharing similar marker profiles were merged into a single cluster, yielding PM4 in the final integrated dataset. Color gradient indicates the expression level while the size of the dot represents percentage of cells showing the expression. (E) Feature plots showing expression of pan-hemocyte markers Hml, Srp, NimC1, and He on the tSNE map, confirming the identity of profiled cells as hemocytes. The blue color gradient indicates the expression level of each gene. Hml is the most prominent hemocyte-specific gene in our dataset. (F) Violin plots showing the cell type-specific expression of pan-hemocyte markers Hml, NimC1, Srp, and He across individual hemocyte clusters. While Hml is broadly and highly expressed in all clusters, NimC1, Srp, and He display more restricted and cluster-specific expression patterns. Supplementary Figure S2. Assignment of hemocyte cluster identities using published marker gene sets (A,B) Feature plots showing expression of Lamellocyte (A [file media-1.pdf]
